# First observation on the predation of a non-arthropod species by a dung beetle species: The case of *Canthon chalybaeus* and the snail *Bulimulus apodemetes*

**DOI:** 10.1371/journal.pone.0258396

**Published:** 2021-10-13

**Authors:** Claudia M. Martín, Andrea del V. Guanuco, Vieyle Cortez, José R. Verdú

**Affiliations:** 1 Instituto de Ecorregiones Andinas (CONICET-UNJu), Jujuy, Argentina; 2 Centro de Investigaciones y Estudios de Diversidad Vegetal (CIEDIVE), Facultad de Ciencias Agrarias, Universidad Nacional de Jujuy, Jujuy, Argentina; 3 Research Institute CIBIO (Centro Iberoamericano de la Biodiversidad) Science Park, University of Alicante, Alicante, Spain; University of California, UNITED STATES

## Abstract

We described, for the first time, a case of predation of a non-arthropod species by a dung beetle species. *Canthon chalybaeus* Blanchard, 1843 kills healthy individuals of the terrestrial snail *Bulimulus apodemetes* (D’Orbigny, 1835) showing an evident pattern of physical aggressiveness in the attacks using the dentate clypeus and the anterior tibiae. The description of this predatory behaviour was complemented with the analysis of the chemical secretions of the pygidial glands of *C*. *chalybaeus*, highlighting those main chemical compounds that, due to their potential toxicity, could contribute to death of the snail. We observed a high frequency of predatory interactions reinforcing the idea that predation in dung beetles is not accidental and although it is opportunistic it involves a series of behavioural sophistications that suggest an evolutionary pattern within Deltochilini that should not only be better studied from a behavioural point of view but also phylogenetically.

## Introduction

Dung beetles (Coleoptera: Scarabaeidae) feed mainly on vertebrate herbivore dung being the main taxonomic group responsible for the recycling of the nutrients it contains. However, in the Neotropical region, necrophagy is also a frequent trophic behaviour in several tribes (e.g. Deltochilini, Phanaeini). In some cases, necrophagy becomes an obligatory condition when the immature stages necessarily require a supply of carrion for their development, as documented in several *Canthon* species [1–3]. In other cases, this condition is optional, being considered the species as copro-necrophagous [1]. Among those strict necrophagous species, only few cases of predation of dung beetles on other arthropods have been observed, being the cases of *Canthon virens* Mannerheim, 1829 and *C*. *dives* Harold, 1868, preying on the leafcutter ant *Atta laevigata* (Smith, 1858), the first and most documented [4–9]. In this case, since both *Canthon* species lack the mouthparts to kill such as cutter mandibles [10], they use the same acute denticles of their clypeus that is used to cut carrion to decapitate ants [e.g. 9]. Another well-documented cases are those of *Deltochilum (Aganhyboma) acropyge* Bates, 1887, *D*. (*A*.) *kolbei* Paulian, 1938 and *D*. (*A*.) *viridescens* Martínez, 1948, which prey on millipedes (Diplopoda). Here too, the kill occurs by decapitation using the clypeus as a ‘cutting weapon’ [11–15]. Other potential cases of predation on diplopods may occur in other Neotropical dung beetles, such as *Canthon morsei* Howden, 1966 [16, 17] or *C*. *aff*. *forcipatus* Harold, 1868 (JRV pers. obs.), as well as in some southern African dung beetles as *Sceliages adamastor* (Le Peletier, 1828), *S*. *granulatus* Forgie and Scholtz, 2002, *S*. *hippias* Westwood, 1844, *Scarabaeus proboscideus* Guárin, 1844, *S*. *satyrus* (Boheman, 1860), *S*. (*Scarabaeolus*) *flavicornis* (Boheman, 1860), *Onthophagus bicavifrons* d’Orbigny, 1902 and *O*. *latigibber* d’Orbigny, 1902 [18–20]. However, although a clear attraction has been observed for the chemical secretions emitted by diplopods, it has not been observed that the dung beetles kill healthy individuals but rather that they are observed on injured, dying or dead diplopods.

**Fig 1:**
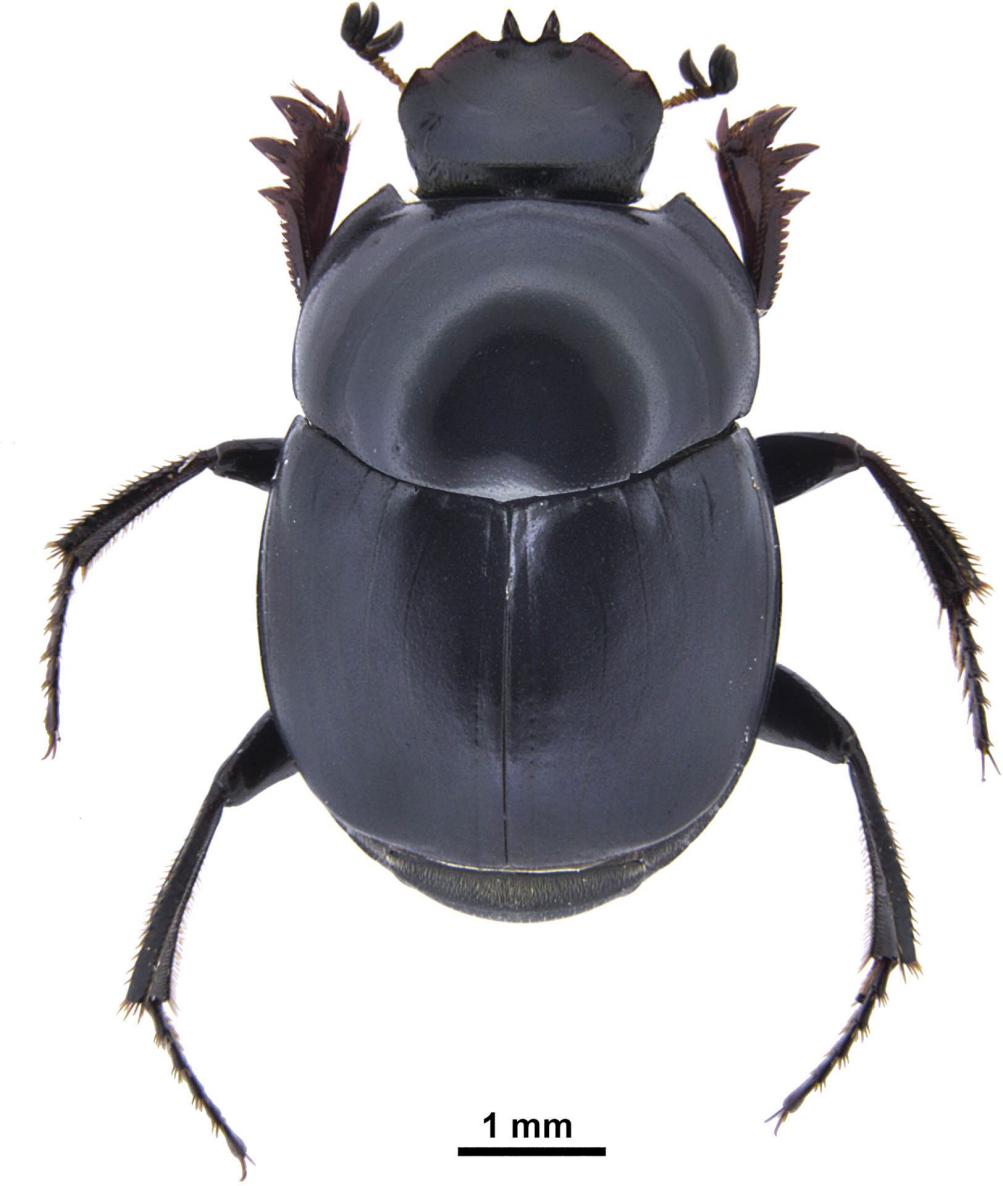
Habitus of *Canthon chalybaeus* showing cutting tools such as the denticles of the clypeus and anterior tibiae.

Here, we described a new case of predation by *Canthon chalybaeus* Blanchard, 1843 (Fig 1), which kills healthy individuals of the terrestrial snail *Bulimulus apodemetes* (D’Orbigny, 1835), being the first reported case of a dung beetle preying on a non-arthropod species. *Canthon chalybaeus* is a relatively small dung beetle, sized 7.5–8.6 mm, known from northern Argentina, southern Bolivia, southern Brazil, Paraguay and Peru [21]. Usually, this species is attracted to carrion baited traps although much less frequently it can be captured in traps baited with human excrement [22]. *Bulimulus apodemetes* is a terrestrial snail 22–29 mm long and 12.7–17.7 mm in maximum diameter, known from northeastern Argentina [23].

As in the other cases explained above, *C*. *chalybaeus* is not an obligate predator but a necrophagous species that has recently been observed exhibiting an evident predatory behaviour on a snail that is significantly larger than it. For this reason, in the present work, a field study was carried out in order to determine what are the ‘weapons’ used by the beetle to kill snails that not only exceeded it several times in size, but also presented a clear defensive characteristic as the shell. Previous studies about the chemical ecology of dung beetles among phylogenetically related species, such as *C*. *conformis* Harold, 1868 (JRV, VC, unpublished data) and *C*. *cyanellus* LeConte, 1859 [24, 25] allowed us to suggest the hypothesis that the death of snails was not only produced by physical aggressions through clypeal denticles as occurs in other species, but that there could be a potential toxic chemical aggression that in a complementary way, would help to kill the snail quickly and more effectively. Thus, in this study we describe in detail the predatory behaviour of *C*. *chalybaeus* on *B*. *apodemetes* under field conditions, providing videographic material that shows each step of the process from the encounter to the burial of the prey. The description of this predatory behaviour is complemented with the analysis of the chemical secretions of the pygidial glands of *C*. *chalybaeus*, highlighting those main chemical compounds that, due to their potential toxicity, could contribute to cause the death of the snail.

## Material and methods

### Study site

Observations took place at Estancia Las Lauras in San Pedro (Jujuy, Argentina) (S 24°34’16.1’’; W 64°39’03.7’’). The altitude of the study area is 995 m a.s.l., with a mean annual temperature from 17.7 to 20.2°C, and the mean annual precipitation of 431–737 mm [26]. The area belongs to the phytogeographic district of the Bosque Chaqueño Occidental [27]. The dominant vegetation type is a xerophilous and deciduous forest of *Schinopsis lorentzii* (Griseb.) Engl., *Libidibia paraguariensis* (D. Parodi) G.P. Lewis, *Gochnatia palosanto* Cabrera, *Geoffroea decorticans* (Gillies ex Hook. & Arn.) Burkart, *Ceiba chodatii* (Hassl.) Ravenna and *Athyana weinmanniifolia* (Griseb.) Radlk.

### Field work description

Observations were carried out with the naked eye, following a “sequence sampling” registering, by means of video recording, all the behaviours under study, in order of appearance. The sample continues until the interaction sequence ends or is interrupted, and the next sample begins with the start of another interaction sequence [28]. The field observations were carried out between the months of February and March of 2021, although the first observation of the interaction was made in April 2015 and confirmed in February 2020. The daily observation period began at 11:00 hours and ended at 16:00 hours, coinciding with the decline in beetle activity.

To calculate the frequency of predation, five plots of 25 m^2^ were delimited and the number of *B*. *apodemetes* predated by the *C*. *chalybaeus* and the number of snails that at no time had any interaction with the dung beetles were counted. The predator-prey mass ratio (PPMR) was also calculated recording the fresh weight of the two species collected in 2021 using a precision balance (resolution: 0.007 g, RCBS Charge Master 1500). PPMR was log-transformed indicating the order of magnitude by which predator individuals are larger than their prey individuals [29].

### Sampling and extraction of chemical compounds from the pygidial glands

We analysed the chemical composition of the pygidial gland secretions of *C*. *chalybaeus* collected at Finca Arroyo del Medio in San Pedro (Jujuy, Argentina) (S 24°27’31.8’’; W 64°41’13.3’’) in December 2015, using the extraction method proposed by [25]. A sample (10 beetles per sample) was collected using a small piece of filter paper that had been cleaned previously with hexane (HPLC grade, Sigma-Aldrich) and placed into a 1.5 ml glass vial with a screw cap (Teknokroma). Volatile compounds were extracted by stir bar sorptive extraction (SBSE) using a freshly conditioned Twister^®^ (stir bar, 0.5 mm thick, 10 mm long, polydimethylsiloxane coating, Gerstel, Mühlheim an der Ruhr, Germany). The PDMS stir bars were pre-conditioned before use by treatment with acetonitrile (HPLC-grade) for cleaning, and conditioned at 250°C for 15 h with a 75 ml/min flow of purified helium. The samples were agitated at 100 rpm, for 6 h at 28°C using a MIR-153 programmable heated and cooled incubator (SANYO Electric Co., Ltd) with an accuracy of 0.2°C. As a control, we put clean filter papers into a glass vial. Three replicates each were performed for the control and the samples. Following extraction, the PDMS stir bar was removed from the glass vial and inserted into the appropriated thermal desorption glass tube (Gerstel 187 mm length × 4 mm I.D., Gerstel GmbH & Co. KG.).

### Chemical identification

Samples were analysed using a thermal desorption system (Gerstel TDS-2) for 10 min at 300°C and with a helium flow rate of 55 ml/min, connected to a gas chromatograph coupled to a mass selective detector (GC-MS). GC-MS was carried out with an Agilent 5973MS coupled with an Agilent 6890GC equipped with a DB-5 capillary column (30 m × 0.25 mm I.D., 0.25 μm film thickness). Helium was the carrier gas (1.4 ml/min constant flow). The oven temperature was programmed for 5 min at 60°C, 5°C/min increase to 250°C, and then held for 10 min. Injector temperature was set at 250°C (Split mode). The MS transfer-line was held at 280°C and the MS Quadropole and MS source temperatures were 150°C and 250°C, respectively. Mass spectra were taken in EI mode (at 70 eV) in the range of 40 to 450 m/z with a scanning rate of 2.65 scans/s. GC-MS data were processed using the MSD ChemStation software (Agilent Technologies). Tentative compound identifications of secretion components were done by comparison of mass spectra in the WILEY and NIST computerized mass spectral library. Retention indices were calculated using a series of linear alkanes C7-C30 (Sigma-Aldrich 49451-U), obtained under the same chromatographic conditions and compared against literature values [30]. Identifications were confirmed by comparison of spectra and retention times with those of authentic standards when these were available. Commercial standards were purchased from chemical suppliers (Fluka, Sigma, Aldrich, Avocado and Acros), with at least ≥ 98% purity, and were run under the same conditions as the samples. The compounds clearly identified (≥ 90% of quality and confirmed by fragmentation patterns analysis) in the pygidial gland secretions were classified in functional groups, with each group was expressed as a percentage of the total content of the compounds.

### Permits

The research presented here adhered to all provincial and national laws. The Mustad family gave us permission to investigate at Estancia Las Lauras. Also, the Dirección Provincial de Biodiversidad de Jujuy–Secretaría de Gestión Ambiental (Jujuy, Argentina) provided permission to conduct the research under permit number 171/2015-DPB.

## Results

### Predation behaviour

**Fig 2:**
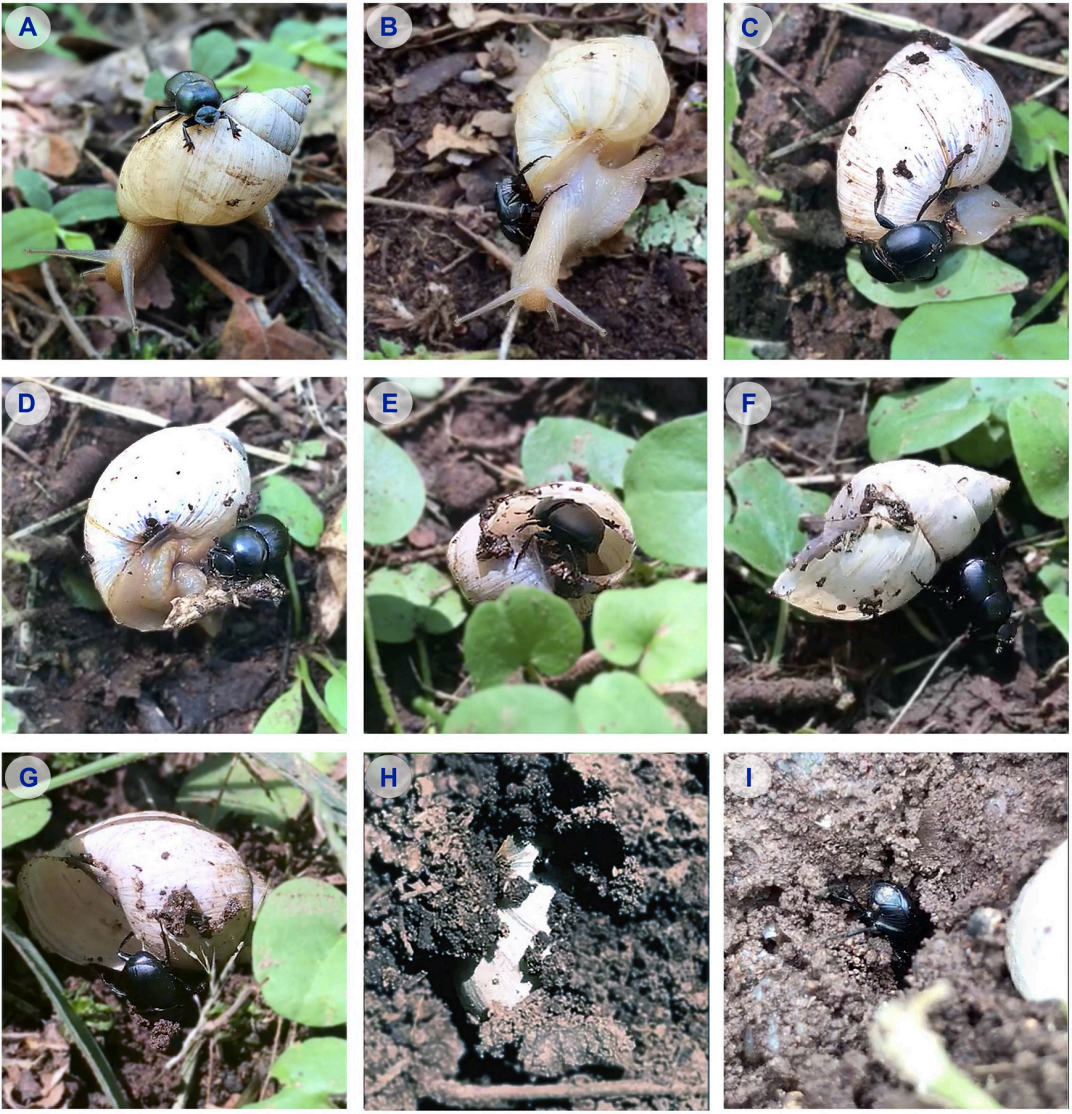
Predation on *Bulimulus apodemetes* by *Canthon chalybaeus*. (A) The beetle climbs up and attaches itself to the shell of the snail. (B) The beetle begins the physical aggression. (C) The beetle frenetically increases its aggressiveness making cuts and lacerations in the soft body of the snail. (D) The snail decreases its mobility and the beetle increases its aggressiveness. (E) During several minutes physical injuries goes on for the necessary time until the snail dies. (F) The beetle rolls the dead snail in search of a suitable place to bury it. (G) The beetle begins to dig into the ground below the snail until it is completely buried. (H) When the snail is buried, the beetle breaks its shell and makes a food ball. (I) The beetle (if male) begins to emit sexual pheromones to attract the female (for more detailed information, see text and S1 Video).

A total of 46 predatory interactions were recorded. The frequency of predation, based on the sampling design described above, showed a percentage of 11.5 ± 6.2%. The observed predatory behaviour always show a distinctive pattern (see Fig 2 and S1–S9 Videos), as follows: (A) A beetle climbs up and attaches itself to shell of the snail, remaining almost motionless for several minutes (10–25 min) while the snail moves along normally. On some occasions, the snail detected the beetle and tried to get rid of it by twisting its body without success. (B) The beetle begins the aggression by injuring the soft body of the snail by making rapid movements with its anterior tibiae and clypeus. Both parts of the body are provided with sharp denticles generally used for cutting meat. The first attack occurs from the shell, affecting mainly the dorsal and right lateral part of the soft body of the snail, with duration of 1.5–3 minutes. (C) The beetle begins a much more aggressive, almost frenetic attack phase, in which the attacks with the clypeus and the contacts with the whole body are intensified. The snail, during this period (3–5 min), stops moving and, although it tries to escape, it begins to partially retract into its shell. (D) As the snail decreases its mobility, the beetle increases its aggressiveness increasing the attacks with the clypeus and the tibiae. (E) This behaviour goes on for the necessary time (4–5 min) until the snail dies. (F) Once the snail is dead, the beetle rolls the snail for a time (5–6 min) in search of a suitable place to bury it. (G) Once the beetle finds a suitable burial place for the snail, it begins to dig into the ground below the snail until it is completely buried (8–10 min). (H) After completely burying it, the beetle breaks the shell and extracts the entire soft body with which it makes a food mass (2–3 min). (I) If the beetle is male, once the construction of the food mass that will serve as a ‘nuptial offer’ is completed, the beetle begins to emit sexual pheromones (around 5 min) to attract the female. If the predation was produced by a female, the behaviour was similar except for the last step in which the female remained buried with the snail after extracting it from the shell. The dung beetle sex-ratio of predation incidences was 2.3:1.0 (male:female).

### Additional observations

**Fig 3:**
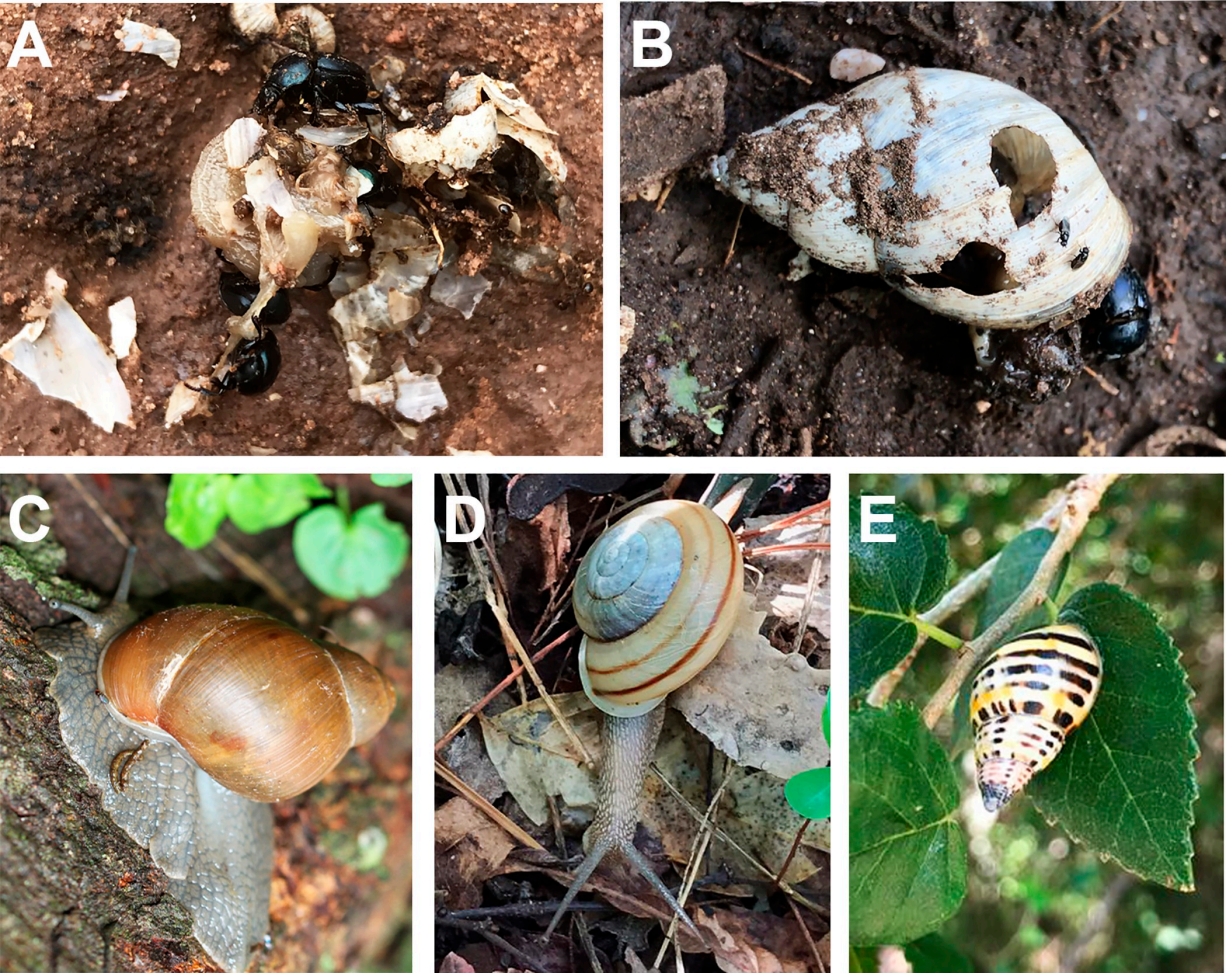
**A:** *Canthon chalybaeus* feeding on carcasses of snails crushed. **B:** *Bulimulus apodemetes* with its shell broken and preyed upon by *C*. *chalybaeus*. **C-E:** Snail species observed in the study area. **C:** *Megalobulimus oblongus musculus*. **D:** *Drymaeus poecilus*. **E:** *Epiphragmophora trigrammephora*.

Sometimes on a few tracks, specimens of *C*. *chalybaeus* were observed feeding on carcasses of snails crushed by the passage of vehicles or big mammals (e.g. tapirs, horses, cows). These cases of necrophagy in the study area were also sporadically observed in *Canthon quinquemaculatus* (Fig 3). In the field, individuals of other species of snails [*Megalobulimus oblongus musculus* (Bequaert,1948), *Drymaeus poecilus* (D’ Orbigny, 1835) and *Epiphragmophora trigrammephora* (D’ Orbigny, 1835)] were also observed but never interacting with *C*. *chalybaeus* (Fig 3).

### Predator-prey mass ratio

The average fresh weight of *C*. *chalybaeus* was 0.030 ± 0.009 g, while the average fresh weight of the predated specimens of *B*. *apodemetes* was 2.590 ± 0.647 g. This notable difference in weights showed a PPMR equal to –1.94.

### Chemical compounds from the secretion of pygidial glands

A total of 8 main compounds were identified in the pygidial glands of *C*. *chalybaeus* by comparing their mass spectra and retention indices with those of available standards (see Table 1). The chemical patterns found in the pygidial secretions included carboxylic acids, phenols, aromatic heterocyclic compounds and a miscellaneous group of compounds. The major components in pygidial gland secretions were indole (25.6%), acetic acid (16.3%), trimethylamine (11.0%), N-(3-methylbutyl) acetamide (6.2%), phenol (5.5%) and butyric acid (5.3%) representing 70% of the total of chemical composition.

**Table 1:** Chemical composition and their potential biological functions of the pygidial gland secretions of *Canthon chalybaeus*.

Compound*	RI^a^	RI^b^	Identified^c^	Functional group	Presumed biological significance	Composition (%)^d^
Trimethylamine	-	-	MS	Miscellaneous		11.0
Acetic acid	-	-	MS	Carboxylic acid	insect repellent^1^; defence^2^	16.3
Butyric acid	-	763	MS	Carboxylic acid	insect repellent^1^	4.7
Isovaleric acid	825	827	MS, ST	Carboxylic acid	insect repellent^1^^,^^3^; defence^4^^,^^5^^,^^6^^,^^7^^,^^8^	3.0
Valeric acid	832	-	MS, ST	Carboxylic acid	defence^9^	4.8
Phenol	977	-	MS, ST	Phenol	defence^2^	5.5
N-(3-methylbutyl) acetamide	1152	-	MS	Miscellaneous	venom^10^^,^^11^^,^^12^^,^^13^^,^^14^	6.2
Indole	1298	1290	MS, RI, ST	Aromatic heterocyclic		25.6

* Compounds listed in order of elution on a polar DB5 capillary column.

^a^Retention indices determined using the homologous series of n-alkanes (C7–C30).

^b^Retention indices obtained using data from the literature [30].

^c^Method of identification: MS, identified by comparison with mass spectra databases; RI, identified by retention indices; ST, comparison with the retention times and mass spectra of available standards.

^d^Relative abundance calculated from GC-MS peak areas.

References

^1^ [31]

^2^ [32]

^3^ [33]

^4^ [34]

^5^ [35]

^6^ [36]

^7^ [37]

^8^ [38]

^9^ [39]

^10^ [40]

^11^ [41]

^12^ [42]

^13^ [43]

^14^ [44].

Possible biological functions of the compounds were compiled in Table 1. Most of the chemicals detected in this study have been reported previously as defensive compounds and venoms in other insect species.

## Discussion

The evident patterns of physical aggressiveness in the attacks and the high frequency of predatory interactions confirm that *C*. *chalybaeus* is an efficient predator of *B*. *apodemetes*. With this interaction, there are six species of dung beetles that certainly prey on other species with a surprising degree of sophistication and hostility. In all these interactions, the use of the clypeal denticles was key to cause injuries or kill the prey. In the other five cases of predation previously described, the prey were ants and diplopods, and the main way of causing death was by decapitation or disarticulation of segments [4, 9, 12]. In *C*. *chalybaeus*, the mode of operation is very different, since the physical aggression through the denticles of the clypeus and the anterior tibiae only causes cuts in the snail integument, so death required the secretions from the pygidial glands whose toxic compounds could be more damaging when penetrating through the lacerations made.

Gastropod feeding (malacophagy) by other Coleoptera families, such as some Carabidae, Lampyridae, Silphidae, Drilidae, Staphylinidae, Dityscidae and Hydrophilidae has been well documented, showing morphological adaptations to kill snails such as elongation of the head, flattening of the head and pronotum, powerful and asymmetrical mandibles, etc. [45–48]. In most cases, the attack and death of the snail is produced by the action of the mouthparts, and in very few cases, as for example in the firefly *Pyrocoelia atripennis*, the use of toxins from midgut extracts to paralyze and kill the snail has been suggested [49].

In dung beetles, and concretely in *Canthon* species, pygidial gland secretions can play an important role in the resource competition, protecting the brood ball and defence it against predators [24, 25]. For this reason, the chemical composition of pygidial gland secretions of some *Canthon* subspecies, such as *C*. *cyanellus cyanellus* and *C*. *femoralis femoralis* (Chevrolat, 1834), comprises a great diversity and of the chemical functional groups including aliphatic and sesquiterpene hydrocarbons, aldehydes, carboxylic acids, fatty acids, monoterpenes, phenols, ketones, sulphur compounds, and a miscellaneous group of molecules [25]. In our study, the chemical patterns found in *C*. *chalybaeus* were very diverse, which included carboxylic acids, phenols, aromatic heterocyclic compounds and a miscellaneous group of compounds (Table 1). Some of these molecules are common allomones with confirmed defensive and repellent functions. For example, indole has been reported in defensive secretions of several beetle species [25, 50–52]; acetic acid has been found frequently in defensive secretions of Carabidae [53–55]; the chemical N-(3-methylbutyl) acetamide, reported for the first time in dung beetles, has previously been found in the venom of Vespinae and Polistinae social wasps [40–42, 56]. Thus, we can suggest that *C*. *chalybaeus* could use their pygidial gland secretions as a chemical weapon in its predation behaviour even if the main role of these compounds are for the defence and protection of brood balls.

This unusual case of predation showed another peculiarity since the PPMR that was observed was surprisingly much lower than that observed in nature, even in host-parasitoid interactions [57]. In general, the body mass of predators is about 100 times larger than that of their prey (PPMR = 2), although marked variations have also been found [29]. At present, data on PPMRs are lacking when predators have a comparable or even smaller body size than their prey, however, in the case of *C*. *chalybaeus*, it has been verified that the variations in the body size of the predator and the prey were of very different orders of magnitude and negative (PPMR = –1.94). An also negative relationship, although in a smaller order of magnitude, was obtained in the interaction between *C*. *viridis* and *A*. *laevigata* (PPMR = –0.96; based on body weights from [58] and [59]), showing that for predatory dung beetles a PPMR around 2 is very far from what is observed in the field. Until more information is available, we suggest that these negative PPMRs may be due to the fact that the main purpose of both predatory *Canthon* species is not only for individual feeding, but also for the provision of food for the larvae, which is why it requires a greater quantity of flesh to construct the brood ball.

We can conclude that this astonishing behaviour reinforces the idea that predation in dung beetles is not accidental and although it is opportunistic it involves a series of behavioural sophistications that suggest an evolutionary pattern within Deltochilini that should not only be better studied from a behavioural point of view but also phylogenetically. Ecological transitions like the ones described here are important to understanding dung beetle evolution and diversification, providing an unusual leap through trophic levels within a typical necrophagous guild.

## Supporting information

S1 Video Videorecording of the predation of *Bulimulus apodemetes* by *Canthon chalybaeus*. Step 1: the dung beetle is attached to the shell of the snail and remains motionless for several minutes.
(MOV)

S2 Video Videorecording of the predation of *Bulimulus apodemetes* by *Canthon chalybaeu*. Step 2: the dung beetle begins the attack by rubbing the tibiae and clypeus on the soft body of the snail.
(MOV)

S3 Video Videorecording of the predation of *Bulimulus apodemetes* by *Canthon chalybaeus*. Step 3: the dung beetle progressively increases its aggressiveness forcing the snail to slow down its movement.
(MOV)

S4 Video Videorecording of the predation of *Bulimulus apodemetes* by *Canthon chalybaeus*. Step 4: the dung beetle immobilizes the snail and continues the aggression for minutes.
(MOV)

S5 Video Videorecording of the predation of *Bulimulus apodemetes* by *Canthon chalybaeus*. Step 5: the dung beetle kills the snail after injuries caused by the action of the clypeus and the anterior tibiae.
(MOV)

S6 Video Videorecording of the predation of *Bulimulus apodemetes* by *Canthon chalybaeus*. Step 6: the dung beetle rolls the snail in search of a suitable place to bury it.
(MOV)

S7 Video Videorecording of the predation of *Bulimulus apodemetes* by *Canthon chalybaeus*. Step 7: the dung beetle, once it finds a suitable place, begins the burial of the snail.
(MOV)

S8 Video Videorecording of the predation of *Bulimulus apodemetes* by *Canthon chalybaeus*. Step 8: the dung beetle partially buries the snail and begins to extract its soft body to separate it from the shell.
(MOV)

S9 Video Videorecording of the predation of *Bulimulus apodemetes* by *Canthon chalybaeus*. Step 9: the dung beetle after getting rid of the empty shell begins to emit sexual pheromones to attract the female.
(MOV)
